# PVDF Sensor Foils Employed to Measure Shear Stress and Temperature of Friction Welding

**DOI:** 10.3390/s20164565

**Published:** 2020-08-14

**Authors:** Wei Zou, Werner Karl Schomburg

**Affiliations:** KEmikro, Campus-Boulevard 30, RWTH Aachen University, 52074 Aachen, Germany; Schomburg@KEmikro.RWTH-Aachen.de

**Keywords:** PVDF sensor, piezoelectric, pyroelectric, shear stress, temperature, friction welding

## Abstract

Friction welding is a popular process for joining metal and polymer work pieces by rubbing them against each other. This way, friction heat is generated in a zone of the faying surfaces, thinner than 1 mm. After cooling down, the heated surfaces establish a tight and strong bond. To improve this joining process, a method is desirable allowing measuring generated temperature and shear stress in the zone between the joining work pieces. Unfortunately, this is a very difficult task because the welding zone cannot be accessed with a sensor without significantly altering the process and thereby the desired measurement results. This paper describes how shear stress and temperature change generated by rubbing polymer pieces in a friction welding machine have been measured between the faying surfaces by employing sensor foils from the piezoelectric and pyroelectric polymer polyvinylidene fluoride (PVDF). This way, heating and cooling rates, pressure rise of the pneumatic system, frequency rise of the starting machine, the duration of starting and stopping, the damping of the vibrations after the drive was stopped, and the stress generated by the pullback of the machine head have been measured. A careful characterization of the sensor was necessary to enable distinguishing the measured voltage due to straining, shearing and temperature change.

## 1. Introduction

**Figure 1:**
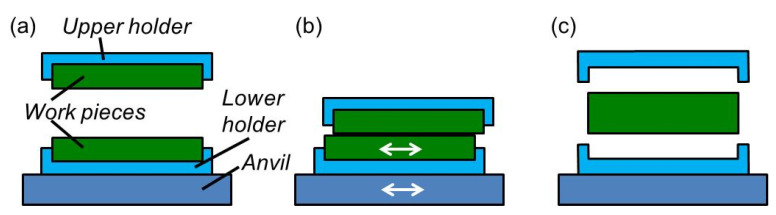
Schematic drawing of friction welding: (**a**) before the process, (**b**) rubbing of the work pieces, (**c**) removing of the welded parts.

Patents on friction welding were already filed in 1942 [1] and 1956 [2]. Friction welding is a joining process employed mainly for metals but also for polymers [3,4,5,6,7,8,9,10,11,12,13]. The surfaces of the work pieces to be joined are mounted at an upper and a lower holder of a friction welding machine (cf. Figure 1a). The machine is closed for instance by driving the upper holder down and the pieces are pressed onto each other (Figure 1b). The upper or lower holder of the friction welding machine is moved in lateral direction generating shear and friction heat between the pieces to be joined. Then, the relative motion is stopped and the work pieces are cooled down while they are still pressed onto each other. During cooling down, a tight and strong bond is established between the work pieces. At the end, the upper holder is pulled back and the bonded sample is removed from the machine (Figure 1c). Friction welding processes are distinguished by the kind of the relative motion of the work pieces such as rotary, circular, linear, orbital or stir friction welding.

The properties of the bond are a function of temperature and stress during the welding process. Especially at the end of the process the relative movement of the work pieces needs to be stopped before cooling down has established a strong bond [14,15]. Otherwise, the bond may be damaged or residual stress in the joining zone is generated weakening the bond. However, it is difficult to measure temperature and stress because they are limited to a zone, less than 1 mm in thickness, enclosed between the two work pieces. Besides this, the high temperature and friction force tend to destroy sensors in between the two surfaces.

To the knowledge of the authors, there is one publication describing the measurement of shear forces during friction welding by acceleration sensors mounted on upper and lower holder of the friction welding machine [16]. Since a measurement of temperature was virtually impossible during friction welding, model calculations were performed estimating the temperature [17,18,19,20,21,22]. This is important for the investigation whether or not the faying surfaces are molten and how the bond is established. However, parameters required for reliable results such as the friction coefficient between the joining pieces at elevated temperatures are not available from direct measurements.

These difficulties also occur when pressure and temperature distribution shall be measured in the process of ultrasonic welding. In recent papers, there is described how temperature and pressure during ultrasonic welding were measured employing thin film thermocouples, and both piezoelectric and pyroelectric sensor foils from polyvinylidene fluoride (PVDF) [23,24,25,26,27,28]. This paper introduces the simultaneous measurement of shear stress and temperature rise generated in PVDF foils by linear and circular friction welding. This way, the influence of process parameters and movement type on the welding process can be studied. Due to the Curie temperature of PVDF, the investigations are limited to parameters at which no weld is established even between polymer pieces. However, the measurements described in this paper allow investigating the behavior of friction welding machines, stopping time and sample cooling rates. Thus, potentials for machine improvements can be recognized.

## 2. Characterization of PVDF Foils

Since sensor foils made from PVDF generate a charge between their surfaces due to straining, shearing and temperature change, these foils need to be characterized and calibrated carefully before these effects can be distinguished from each other and measurements can be evaluated.

**Figure 2:**
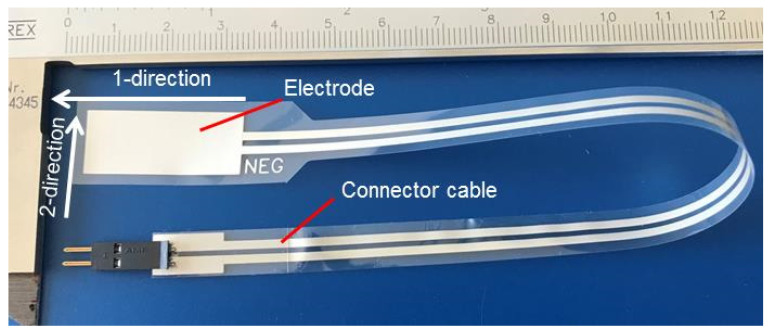
PVDF sensor foil.

The PVDF sensor foils employed for the work described in this paper are called FDT1-028K and were purchased from TE Connectivity (Schaffhausen, Switzerland). The authors could not find any information on the piezoelectric and pyroelectric properties provided by the vendor. Figure 2 shows one of these foils. Such foils are fabricated by extrusion. As a consequence, the sensor foils show anisotropic properties. From the measurements described below, it is recognized that the direction of extrusion is the one denominated as 1-direction in Figure 2. The direction normal to the electrodes is called the 3-direction. In the fabrication process of the sensor foils, a large electrical field had been applied aligning electrical dipoles inside of the polymer. The 2-direction is perpendicular to the other ones and it is also marked in Figure 2.

The electrodes fabricated by the deposition of a silver layer on the PVDF show a smaller area than the sensor foil. In Table 1 the dimensions of sensor foil and electrodes are shown. The entire sensor foils are embedded into an insulating varnish, therefore, the thickness of the entire foil is larger than the PVDF.

### 2.1. Young’s Modulus

Since the PVDF foils have been fabricated by extrusion, their Young’s modulus is a function of direction. Young’s modulus was measured with a tensile testing machine with foils cut in length to 16 mm to obtain comparable dimensions for measurements in 1- and 2-direction. Since it is difficult to measure force and stretching of such thin polymer foils, they were stretched until a force of more than 5 N was measured and the measurement of the extension of the foils was started then. As a consequence, no measurements at smaller strain have been taken, but the slope of the measured curves was employed to calculate Young’s modulus of the foils.

The slopes of the curves measured in 1- and 2-direction have been determined from five measured curves each. Mean value and standard deviation of the slopes are 184.1 ± 0.3 N/mm and 158.2 ± 1.2 N/mm, respectively, corresponding to Young’s moduli of 3 and 2.6 GPa, respectively. The absolute measurement errors of the Young’s moduli are on the order of 10% due to the uncertainty of the thickness measurement. Besides this, the moduli of PVDF foils differ from each other on the order of 15%. It was virtually impossible to perform all measurements described in the following with the same foil. Therefore, an absolute measurement error on the order of 15 to 20% is assumed because the sensor foils employed for the calibration are of the same type and dimensions but not identical to those used for the friction welding experiments.

The Young’s modulus of the PVDF foils was also measured without electrodes and varnish. To achieve this, the varnish was wiped away with acetone. The resulting thickness of the foils and former distance h_E_ of the electrodes was measured to be approximately 30 µm. Since polymer foils tend to creep, three measurements were performed in each direction always employing a new foil. The Young’s moduli in 1- and 2-direction, determined this way, are 3.0 ± 0.1 GPa and 2.42 ± 0.05 GPa, respectively. However, due to the uncertainty in the measured thickness of the foils, the absolute measurement error is on the order of 10%. The fact, that Young’s modulus is larger in 1-direction shows that this is the direction of extrusion because Young’s modulus of polymer foils is increased by plastic deformation due to stretching.

### 2.2. Electrical Measurements

**Figure 3:**
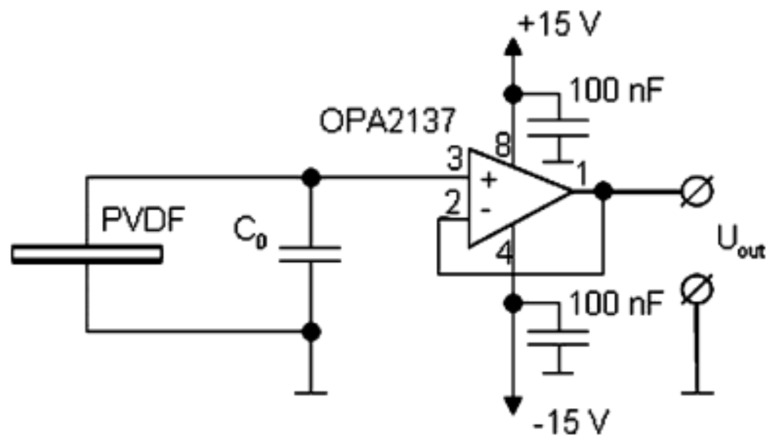
Voltage follower employed to measure the charge generated by a PVDF foil, reprinted from [29] with permission from Elsevier.

The electrical capacity of a PVDF foil is comparatively small and a charge amplifier or a voltage follower needs to be employed to avoid discharging by the measurement (Figure 3) [29,30]. Charge amplifier and voltage follower have a large input impedance of more than 1 GΩ avoiding discharging of the PVDF foil. In the work described in this paper, a voltage follower was used. The output voltage of the circuit was then fed into the analog to digital converter DAQ-Box NI USB-6251 from National Instruments (Austin, TX, USA) with a sample rate of 100 kHz and the digital signal was recorded.

The input voltage of the amplifier employed is limited to 10 V, but in some experiments much larger voltages had been generated on the PVDF. Therefore, for some measurements a capacitor C_0_ was added to the circuit parallel to the PVDF foil distributing the generated charge on a larger capacity and reducing the voltage. Therefore, in the following the total charge Q_3_ generated is calculated from the voltage *U*_m_ measured between the electrodes of a PVDF foil as:

(1) $$Q_{3}=\left(C_{S}+C_{0}\right)\;U_{m}$$

where C_S_ is the capacity of the sensor foil plus the inlet capacity of the amplifier. For most experiments described here, C_S_ was measured with the multi meter 878B from BK Precision (Yorba Linda, CA, USA) at 2 kHz to be 1.37 nF. The capacity C_S_ of the PVDF sensor foil with area A_E_ and distance h_E_ of its electrodes is defined as:

(2) $$C_{S}=\varepsilon_{0}\;\varepsilon_{r}\;\frac{A_{E}}{h_{E}}\;\Rightarrow \;\varepsilon_{r}=\frac{C_{S}\;h_{E}}{\varepsilon_{0}\;A_{E}}$$

where ε_r_ and ε_0_ = 8.9 × 10^−12^ A s/(V m) are relative and absolute permittivity, respectively. With Equation (2) and the dimensions shown in Table 1, the relative permittivity was calculated to be 13.3.

### 2.3. Piezoelectric Effect

The piezoelectric effect of PVDF was first described in 1969 [31]. PVDF is a semi-crystalline thermoplastic polymer and its β-phase is piezoelectric if it has been stretched for several times of its original length and polarized by an electrical field on the order of 30 V/µm near to its crystal melting temperature [31]. If a piezoelectric PVDF foil is strained, electrical charges are rearranged resulting in a voltage which can be measured.

If a body is bent, it is stretched on one side and compressed on the opposite side. Piezoelectric PVDF sensor foils generate charges on their electrodes if they are strained. Therefore, by bending a PVDF foil, there is generated as much stretching as compression, and as a consequence no charge is generated when they are only bent. But when PVDF sensor foils are stretched along their neutral fiber, charges are generated and a voltage U_m_ is measured. The electrical charge density D_3_ on the electrodes is a function of the stress acting in the directions in which the foil had been extruded *σ*_1_, perpendicular to extrusion *σ*_2_, and normal to its electrodes *σ*_3_ (see for instance [32]):

(3) $$D_{3}=d_{31}\;\sigma_{1}+d_{32}\;\sigma_{2}+d_{33}\;\sigma_{3}+\varepsilon_{0}\;\varepsilon_{r}\;E_{el}$$

where *d*_31_, *d*_32_, *d*_33_ and E_el_ are the piezoelectric moduli of PVDF and the electrical field in the foil. The electrical charge Q_3_ on the electrodes is obtained from the above equation by multiplying with the area A_E_ of the electrodes, the stresses are expressed as the forces *F*_1_, *F*_2_ and *F*_3_ divided by the corresponding cross-sections and the electrical field is the voltage U_m_ divided by the distance h_E_ of the electrodes. Besides this, it needs to be taken into account that a capacity C_0_ may be included in the circuit parallel to the sensor foil as shown in Figure 3:

(4) $$Q_{3}=d_{31}\;\frac{A_{E}}{h_{F}\;b_{F}}\;F_{1}+d_{32}\;\frac{A_{E}}{L_{F}\;h_{F}}\;F_{2}+d_{33}\;\frac{A_{E}}{L_{F}\;b_{F}}F_{3}+\left(\frac{\varepsilon_{0}\;\varepsilon_{r}\;A_{E}}{h_{E}}+C_{0}\right)\;U_{m}$$

In Equation (4), the terms in the parenthesis are the capacities of the sensor foil and C_0_. In this equation, it was assumed that the stress of the PVDF and the entire sensor foil with varnish are the same because their Young’s moduli are very similar as shown above.

If the electrodes are short-circuited for a moment, the charge is zero. If in the following time the forces acting on the PVDF foil are changing, the voltage U_m_ measured between the electrodes is found by solving Equation (4). If the capacity parallel to the capacity C_S_ of the PVDF foil is zero (C_0_ = 0), it is obtained:

(5) $$\begin{array}{ll} U_{m} & =\frac{d_{31}}{\varepsilon_{0}\;\varepsilon_{r}}\;\frac{h_{E}}{h_{F}\;b_{F}}\Delta F_{1}+\frac{d_{32}}{\varepsilon_{0}\;\varepsilon_{r}}\;\frac{h_{E}}{L_{F}\;h_{F}}\Delta F_{2}+\frac{d_{33}}{\varepsilon_{0}\;\varepsilon_{r}}\;\frac{h_{E}}{L_{F}\;b_{F}}\Delta F_{3}\\ & =g_{31}\;\frac{h_{E}}{h_{F}\;b_{F}}\Delta F_{1}+g_{32}\;\frac{h_{E}}{L_{F}\;h_{F}}\Delta F_{2}+g_{33}\;\frac{h_{E}}{L_{F}\;b_{F}}\Delta F_{3} \end{array}$$

where the quantities *g*_31_, *g*_32_, and *g*_33_ are the piezoelectric moduli of force measurements. L_F_, b_F_ and h_F_ are length, width and thickness of the PVDF foils, respectively.

When Equation (5) is derived from Equation (4), the sign of the measured voltage is negative and not positive as shown in the equation. However, the sign of the measured voltage is anyway a function of the polarization of the PVDF, the poling of the electrical contacts to the foil, and the kind of the amplifier employed. Therefore, the sign of U_m_ was not taken into account here.

**Figure 4:**
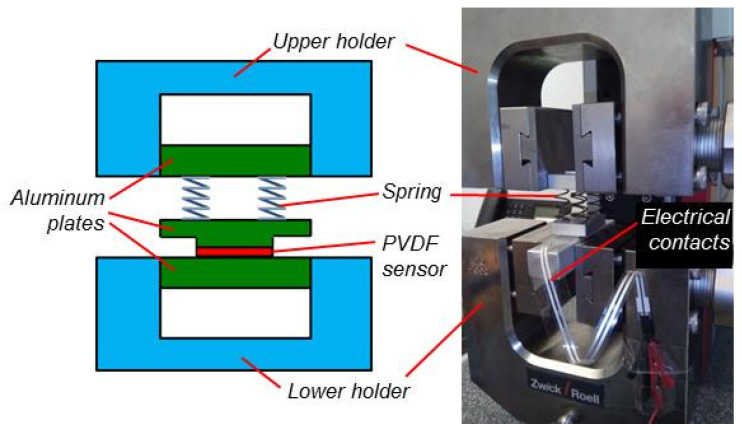
Tensile testing machine equipped with springs to measure the relationship between voltage and force in 3-direction. Left: Schematic cross-section not to scale; right: Photo.

The relationship between exerted force and generated voltages was measured with a tensile testing machine. For these measurements, no capacity C_0_ was employed parallel to the capacities of PVDF foil and amplifier. To measure the voltage as a function of the force acting in polarization direction (3-direction), four springs were mounted between an aluminum plate clamped by the upper holder of the tensile testing machine and a second aluminum plate (Figure 4). On the lower side of the second plate, there was a milled mesa with the same lateral dimensions as the electrodes on the PVDF foil. The aluminum plate was glued onto the electrode and the opposite side of the sensor foil was glued onto a third aluminum plate clamped by the lower holder of the tensile testing machine. This way, the force was rising slower with the movement of the machine allowing more sensitive measurements. Measurements in 1- and 2-direction were performed in the usual way by clamping the PVDF foil in the tensile testing machine.

**Figure 5:**
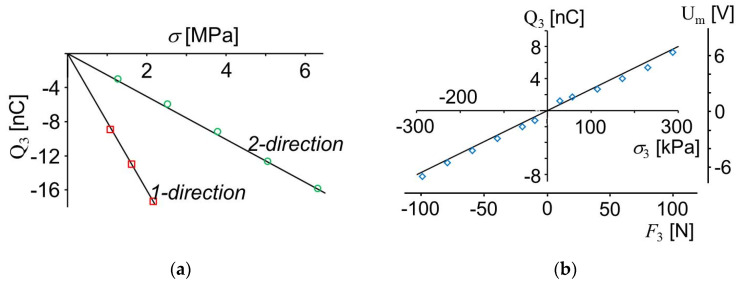
Voltage measured with a PVDF sensor and charge generated as a function of force and pressure exerted in (**a**) 1- and 2-direction, and (**b**) normal to the sensor, in 3-direction.

The electrical charge generated was calculated from the measured voltages by Equation (1) and the stresses σ_1_, σ_2_ and σ_3_ were calculated by dividing the forces by the corresponding cross-sections resulting in the graphs shown in Figure 5. The sign of the voltage change measured is also a function of the polarization of the PVDF and the amplifier employed. Therefore, all measurements were performed with the same electronics and the same poling. The measured voltage as a function of the force acting on the PVDF foil is different if it is stretched in 1- or 2-direction. In both cases voltage and charge are proportional to force and stress.

For the measurements shown in Figure 5, *g*_31_, *g*_32_ and *g*_33_ were calculated by solving Equation (5). Length and width of electrodes and sensor foil are different. The dimensions are shown in Table 1. Besides this, the slopes of the curves shown in Figure 5 were calculated indicating the ratios of generated charge Q_3_ and stress. These results are shown in Table 2. By solving Equation (5) using the relative and absolute permittivity, *d*_31_, *d*_32_ and *d*_33_ were calculated.

If a PVDF foil is pressed in the direction of its thickness, the measured voltage change is also proportional to the force and with the same sign compared to stretching in lateral direction although the direction of the load is different (Figure 5b). Therefore, *g*_33_ has the opposite sign as *g*_31_ and g_32_. As usual, in Table 2 *g*_33_ is shown with a negative sign and *g*_31_ and *g*_32_ are shown positive.

Shearing a piezoelectric PVDF foil also results in an electrical charge generated on its electrodes [33]. To include this effect into the calculations, Equation (3) is extended by the shear stresses *σ*_4_ and *σ*_5_ that occur when a PVDF sensor foil is exerted to shear forces *F*_4_ and *F*_5_ in 1- and 2-direction, respectively:

(6) $$D_{3}=d_{31}\;\sigma_{1}+d_{32}\;\sigma_{2}+d_{33}\;\sigma_{3}+d_{34}\;\sigma_{4}+d_{35}\;\sigma_{5}+\varepsilon_{0}\;\varepsilon_{r}\;E_{el}$$

The shear stresses are the shear force divided by the area of the PVDF foil. Equation (4) correspondingly is extended by two terms:

(7) $$\begin{array}{l} Q_{3}=d_{31}\;\frac{A_{E}}{h_{F}\;b_{F}}\;F_{1}+d_{32}\;\frac{A_{E}}{L_{F}\;h_{F}}\;F_{2}+d_{33}\;\frac{A_{E}}{L_{F}\;b_{F}}F_{3}\\ +d_{34}\;\frac{A_{E}}{L_{F}\;b_{F}}F_{4}+d_{35}\;\frac{A_{E}}{L_{F}\;b_{F}}F_{5}+\left(\frac{\varepsilon_{0}\;\varepsilon_{r}\;A_{E}}{h_{E}}+C_{0}\right)\;U_{m} \end{array}$$

**Figure 6:**
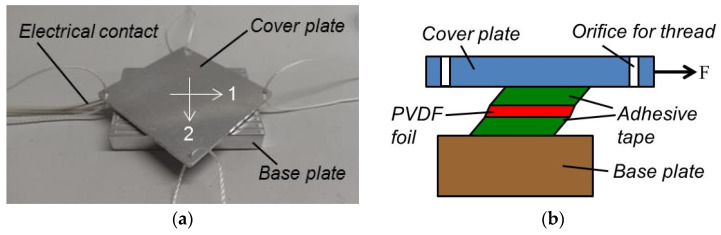
Set up for measuring the voltage generated by shearing a PVDF foil as a photo with 1- and 2-direction marked (**a**) and a schematic drawing (**b**) (not to scale).

The voltage generated due to shear stress was measured by gluing a PVDF foil with 3 M 9088 double sided tape, 210 µm in thickness, between a base plate fixed on a table and a square cover plate from aluminum, 2 mm in thickness (cf. Figure 6). At the corners of the cover plate, there had been drilled orifices through which threads were mounted. At the free end of these threads, there was mounted the force measuring device PCE-FG from PCE Instruments (Meschede, Germany). According to the specifications given by the vendor, the resolution of this force measurement device is 0.01 N with an error limit of up to 0.05% in the range of 0 to 50 N. It stores the maximum force measured and the maximum voltage recorded was measured with the circuit shown in Figure 3 and an oscilloscope. The cover plate was aligned such that the PVDF foil was sheared in 1- or 2-direction by fixing the force measuring device at the corresponding thread. The device was pulled parallel to the table surface in the desired direction relative to the PVDF foil and measured force and voltage were recorded.

**Figure 7:**
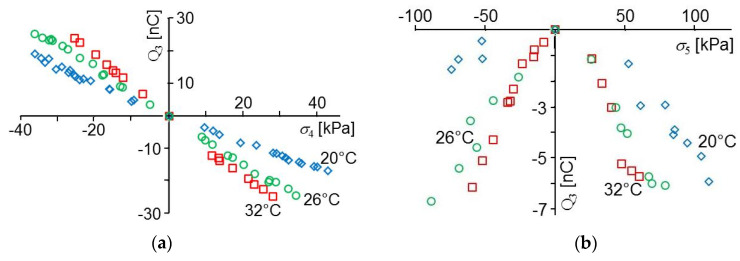
Charge Q_3_ calculated with Equation (1) from the measured voltage U_m_ as a function of the shear stress calculated from the measured force: (**a**) shear *σ*_4_ in 1-direction and (**b**) *σ*_5_ in 2-direction.

The charge Q_3_ calculated with Equation (1) from the measured voltage U_m_ is shown in Figure 7 as a function of the shear stresses *σ*_4_ and *σ*_5_ calculated from the measured forces. The capacity C_0_ was 9.6 nF in this case. The generated charge due to shear stress is a function of both the direction in which the PVDF foil is sheared and temperature.

The sign of the measured charge and voltage is changing if the shear in the orientation of the foil extrusion (1-direction) is changing direction and the characteristic curve shown in Figure 7a is near to a straight line. Unlike this, in the orientation perpendicular to foil extrusion (2-direction), the sign is not a function of the direction of shear and the characteristic curves appear to be like parabolas (cf. Figure 7b). The sign of the signal is the same as if the temperature would be raised and opposite to the sign obtained when the sensor foil is compressed in normal direction or stretched in lateral direction.

It was difficult measuring the small charges generated in 2-directions, and as a consequence, the measured data show a large scatter. In all measured cases the generated charge measured in 2-direction was at least a factor of 10 smaller than the charge obtained with the same shear stress in 1-direction.

The charge generated by shear stress is a function of temperature. The estimated slopes Q_3_/*σ*_4_ of the curves shown in Figure 7a and the piezoelectric moduli *d*_34_ are shown in Table 3 for three temperatures. There is no sign shown in the table because it is unclear in what direction a shear results in a positive charge. A modulus *d*_35_ cannot be shown because the characteristic curves in Figure 7b are not linear.

### 2.4. Pyroelectric Effect of PVDF Foils

Besides piezoelectric, PVDF foils are also pyroelectric [30,34]. That means, if the temperature changes, there are also generated charges on the electrodes. A temperature rise corresponds to a voltage change with the opposite sign as observed with the same measurement circuit when the foil is tensile strained in lateral direction or compressed in polarization direction. The pyroelectric effect was calibrated by placing the PVDF foils on a self-made thermo plate with an electrical temperature control. Onto the PVDF sensor foil, there was laid a sponge for thermal insulation and onto the sponge a weight of approximately 1 kg to ensure a good thermal contact between heating plate and PVDF sensor foil. The PVDF foil was discharged when the plate was at room temperature (24 °C). Then the heating plate was heated up to a certain temperature within less than 1 min and the voltage change U_m_ generated by the elevated temperature was noted. This measurement was repeated for different end temperatures always starting with a heating plate at room temperature.

**Figure 8:**
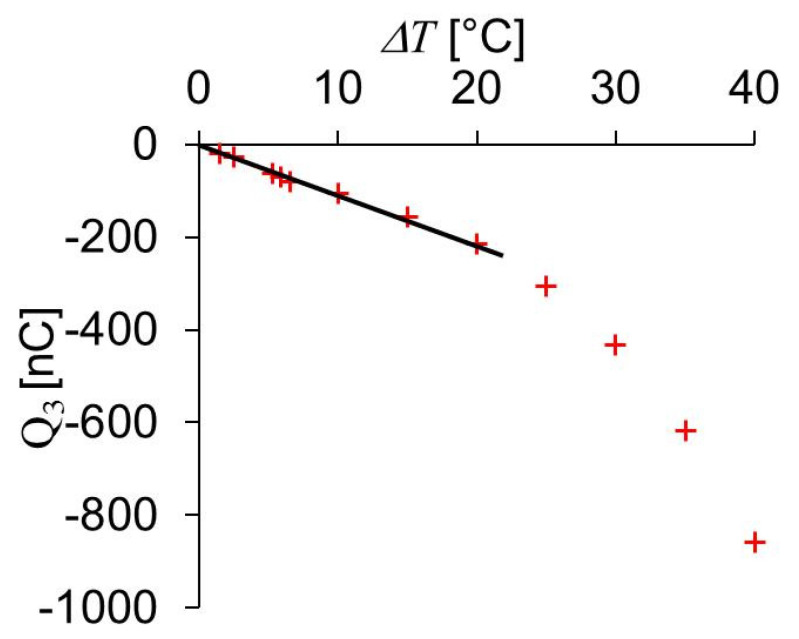
Charge Q_3_ generated by the pyroelectric effect of a PVDF foil as a function of temperature change *ΔT*.

The charge Q_3_ calculated from U_m_ with Equation (1) as a function of temperature change is shown in Figure 8 for one of the four PVDF foils employed. The capacitor C_0_ shown in Figure 3 needed to be changed during the experiments avoiding too large voltages at the amplifier input. Up to 10 °C temperature change *ΔT*, C_0_ was 9.6 nF and at higher temperatures 97 nF. Up to 20 °C, the data shown in Figure 8 can be approximated by a linear curve with a slope of −10.7 nC/°C corresponding to −30.7 µC/(K m^2^).

## 3. Measurement of Shear Stress and Temperature

Measurements were performed both at a linear and at a circular friction welding machine. Both types of friction welding machines press the parts to be welded onto each other and then rub them by moving them relative to each other in lateral direction. Linear welding machines generate a linear back and forth movement, and circular friction welding machines generate movements in circles with diameters of up to a few millimeters.

### 3.1. Linear Friction Welding Machine

**Figure 9:**
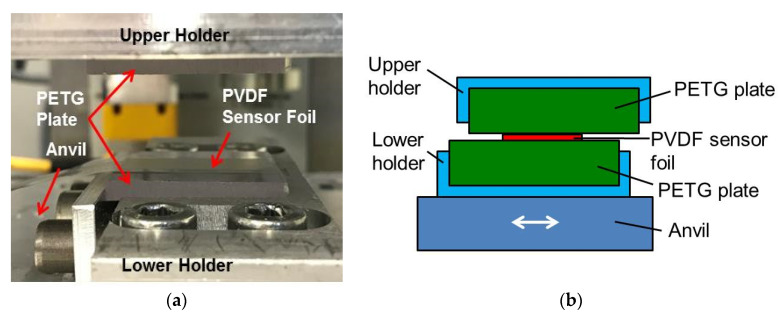
Measurement set-up in the linear friction welding machine shown as (**a**) a photo with a lifted upper holder and (**b**) a schematic cross-section during welding (not to scale).

A lower holder was mounted onto the movable anvil of the linear friction welding machine M-112H from Branson (Dietzenbach, Germany; Figure 9a). The anvil of this machine together with a lower holder and a square plate from the polymer polyethyleneterephthalate glycol (PETG), 31 mm and 4 mm in edge length and thickness, respectively, can be moved back and forth by an electromagnetic drive. It was expected that this movement would be performed as a sine like function of time *t* at frequency f with adjustable amplitude A:

(8) x=A sin(2 π f t)

Another PETG plate with the same dimensions was fixed into an upper holder rigidly fastened to the machine top. To prevent damaging the sensor foils, a comparatively small vibration amplitude of 0.2 mm was applied. Welding time, frequency and holding time were set to 1.5 s, 235 Hz and 1 s, respectively.

**Figure 10:**
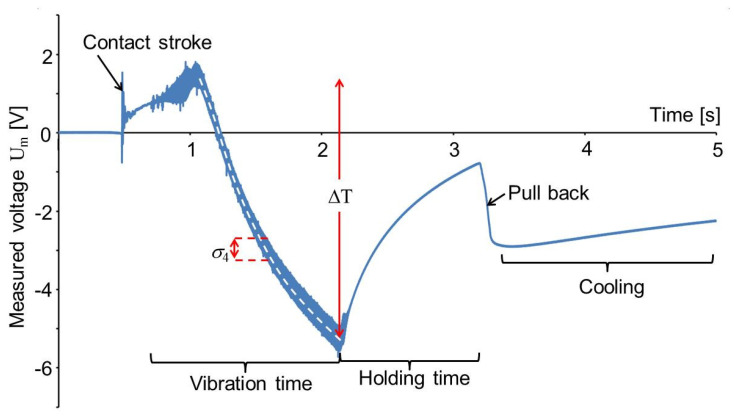
Voltage measured as a function of time by a PVDF sensor foil in a linear friction welding machine.

The pressing force was not recorded by the machine but the pressure difference between the pneumatic cylinder and the environment was measured to be only 100 kPa. The force, generated this way, was acting on the area of the sensor foil and the PETG plate. The sensor foils were placed with their extrusion direction parallel to the movement direction of the linear friction welding machine as shown in Figure 9a. For the measurements, a capacity C_0_ of 9.6 nF was mounted parallel to the PVDF foil to avoid a too large voltage at the inlet of the amplifier (cf. Figure 3). The voltage measured as a function of time is shown in Figure 10. It was recorded with a sampling rate of 100 kHz.

**Figure 11:**
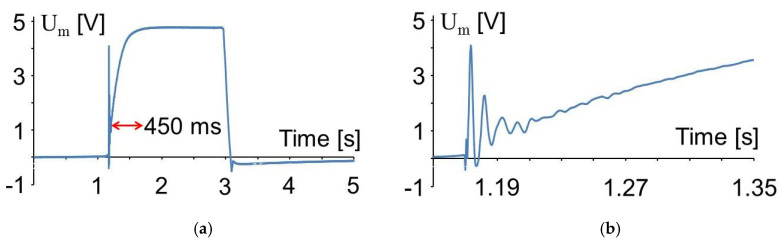
Voltage measured similar to Figure 10*,* but without anvil vibrations (**a**) and time range around the touch of the machine top (**b**).

Some vibrations are observed after the first touch of the machine top. This is understood with an experiment without vibrations of the anvil. In Figure 11a, there is shown a measurement similar as in Figure 10 with C_0_ = 9.6 nF, but without any vibrations of the anvil. The top of the friction welding machine was driven down onto the PVDF foil by the pneumatic drive of the friction welding machine. The enlarged time scale in Figure 11b shows that there are some vibrations with a frequency of approximately 100 Hz after the contact stroke of the machine top. Similar vibrations have been observed when an aluminum plate, 4 mm in thickness, was placed onto the PVDF foil and struck with the handle of a screwdriver. Therefore, the observed vibrations are attributed to the acoustic signal generated by the stroke.

After the stroke, the measured signal in Figure 11a is rising to a constant voltage of 4.76 V. According to Equation (1), this corresponds to a charge of Q_3_ = 54 nC. From Equation (4) and Table 2 the pressure *σ*_3_ on the foil is derived now:

(9) $$\sigma_{3}=\frac{Q_{3}}{d_{33}\;A_{E}}=\;1.9\;\mathrm{MPa}.$$

The rise of the signal is attributed to the filling of the pneumatic cylinder pressing the machine top down onto the anvil. This process lasted approximately 450 ms in this case with a pressure difference of 300 kPa over the valve opening the air flow into the pneumatic cylinder. Measurements at 100 kPa and 500 kPa yielded 530 and 440 ms, respectively.

**Figure 12:**
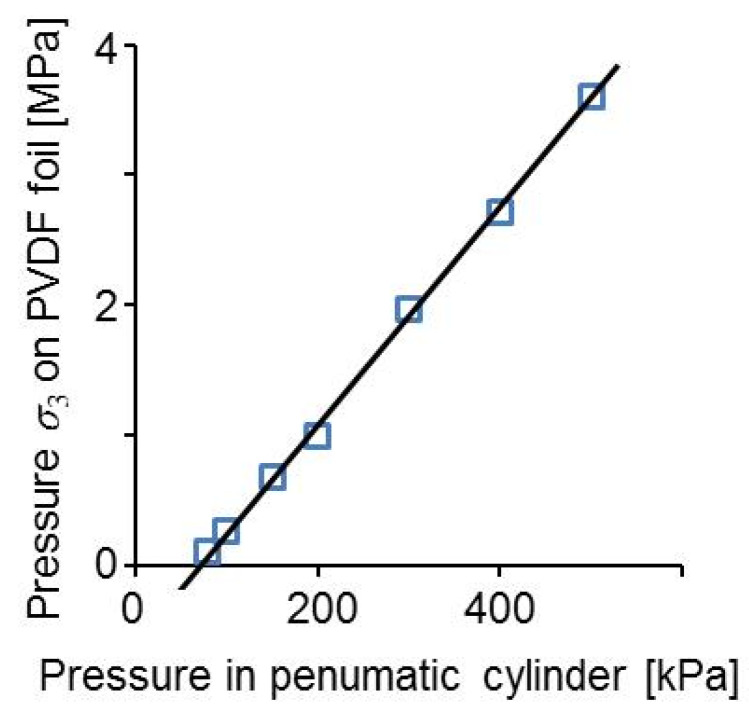
Pressure σ_3_ on the PVDF foil as a function of the pressure in the pneumatic cylinder of the friction welding machine.

The interrelationship between the end pressure in the pneumatic cylinder of the friction welding machine and the pressure on the PVDF foil calculated with Equation (9) is shown in Figure 12. From this figure, it is seen that the pressure load applied in the experiment shown in Figure 9 was *σ*_3_ = 260 kPa corresponding to 100 kPa in the pneumatic cylinder of the friction welding machine.

Two hundred ms after the contact stroke, the anvil together with lower holder und PETG plate started vibrating and generated a periodic change of the voltage. It is observed that the friction welding machine needed 230 ms to arrive at a constant vibration frequency of 235 Hz and an amplitude of 263 mV corresponding to 3.0 nC. From Equation (7) the amplitude A_4_ of shear vibrations is obtained:

(10) $$A_{4}=\frac{Q_{3}}{d_{34}\;A_{E}}=\;6.6\;\mathrm{kPa}.$$

As described above, the maximum pressure in the pneumatic cylinder was reached 530 ms after the contact stroke. Therefore, at 1.1 s in Figure 10 the measured voltage does not rise any more due to an increasing pressure in the pneumatic cylinder. The voltage decrease superimposed to the periodic voltage change in the following time is due to the rising temperature of the PVDF sensor foil generated by friction heat. Some friction heat is expected to be generated also before 1.1 s in Figure 10 and the voltage rise due to the increasing pressure σ_3_ on the PVDF foil will be superimposed with a voltage decrease due to a rising temperature. Therefore the temperature rise (ΔT = 7.1 °C) calculated from the voltage difference (−6.75 V) and charge change (−76.3 nC) as shown in Figure 10 is a lower limit of the real temperature of the PVDF foil. It would have been better to wait another half second with the start of the anvil movements, but when these measurements were performed the interpretation of the signal was not yet clear and the data have been taken as shown in Figure 10.

In general, heating and cooling of a body by heat conduction can be described by an exponential function:

(11) $$T(t)=T_{\infty }+T_{0}\;\exp\left(-\frac{t}{\theta }\right)$$

where (T_0_ + T_∞_) is the temperature before the change starts, T_∞_ is the temperature asymptotically approached, *t* is the time, and θ is a time constant describing how quick the temperature change takes place.

The dashed, white curve shown in Figure 10 has been adapted to the measured data employing Equation (11) with θ = 650 ms. The same way, the cooling in the holding time and after the pull back of the upper part of the welding machine have been determined to be θ = 550 ms and 3.5 s, respectively. The slower cooling after the pull back is attributed to the fact that the heat can dissipate also into the machine top before the pull back and only into the anvil of the machine afterwards. Besides this, the PVDF after the pull back is no longer pressed onto the anvil and the thermal contact may be reduced.

The pull back is performed in 100 ms and generates a voltage change of −2 V corresponding to *σ*_3_ = 810 kPa. The voltage change (+1.64 V) generated by the pressing force at the start of the process is smaller than the one due to the pull back of the holder. The difference of −360 mV is attributed to an adhesion force between PVDF and the upper polymer foil.

**Figure 13:**
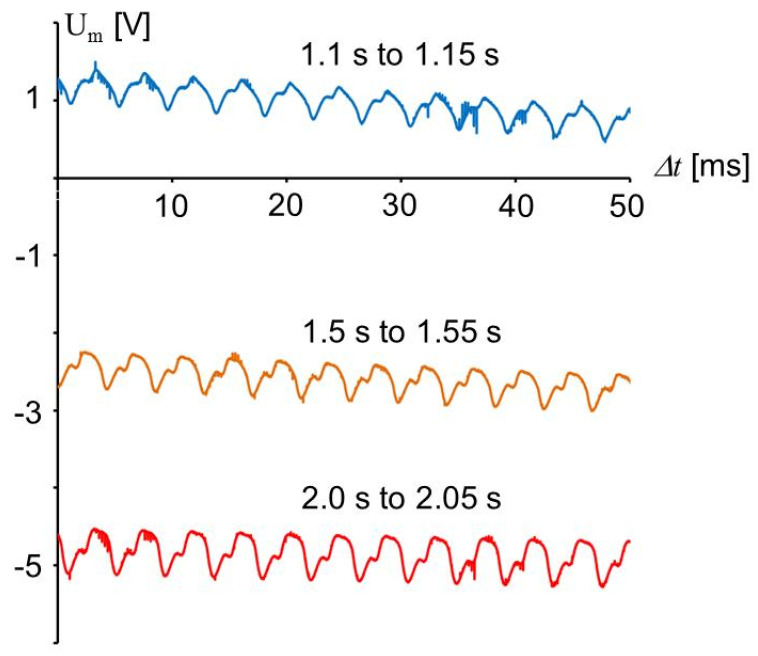
Parts of the signal shown in Figure 10 at different times shown with larger resolution. The corresponding time range from Figure 10 is shown next to the curves.

Figure 13 shows three time ranges of the signal in Figure 10. The shear stress is not a pure sine function and the amplitude is increasing with time by approximately 30%. The increase of the amplitude can be attributed to the fact that the modulus *d*_34_ is increasing by 36% from 20 °C to 26 °C (cf. Table 3). The difference of the signal from a sine function may indicate a not sine like movement of the machine, but this needs to be interpreted with care because other effects have a similar influence on the signal.

**Figure 14:**
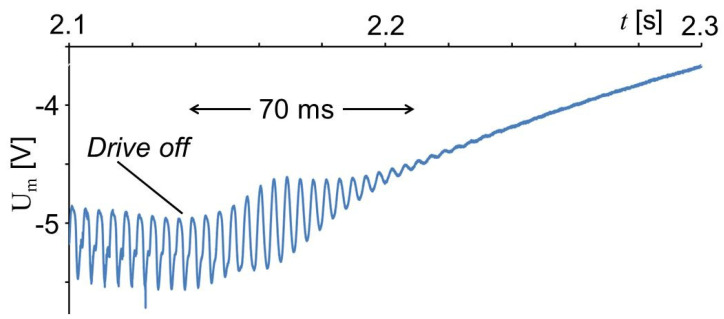
Stopping range of the signal shown in Figure 10.

Figure 14 shows the range of the signal shown in Figure 10 at the end of the anvil vibrations. When the drive is stopped, the measured shear shows the shape of a sine vibration. This could be interpreted as an indication that there is some mechanical effect of the drive, however, it cannot be excluded also that the area of the sensor foil acts like an antenna receiving an electrical signal from the drive.

As already mentioned in the introduction, for friction welding, it is important that the relative movements of anvil and sample stop quickly to ensure a homogeneous bond. The weld strength was 60% larger if the relative movement of the work pieces to be joined stopped in 50 ms instead of 750 ms [14,15]. In Figure 14, it is recognized that the stopping time was 70 ms.

**Figure 15:**
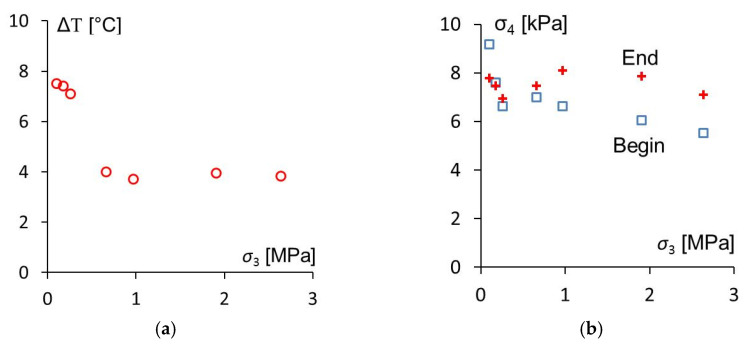
Temperature change ΔT (**a**) und shear stress σ_4_ (**b**) near the beginning and end of the vibrations as a function of the pressure *σ*_3_ compressing the samples.

Temperature change ΔΤ and shear stress σ_4_ were measured as described above from measurements similar as the one shown in Figure 10, but as a function of the pressure *σ*_3_ compressing the sample during the vibrations of the anvil. *σ*_3_ has been determined from Figure 12 and the pressure in the pneumatic cylinder. The results of these measurements are shown in Figure 15. All these measurements were performed with the same PVDF sensor foil.

The measured shear stress at the begin of the vibrations (600 ms after the contact stroke in Figure 10) at a pressure of 105 kPa was about 66% larger than at 2.6 MPa. The average measured shear stress at the end of the vibration (1.6 s after the contact stroke) was 6.9 ± 1.1 kPa.

The temperature raise had its maximum of 7.5 °C at a pressure of 105 kPa. At larger pressures the temperature raise was smaller indicating that there may be an optimum pressure to generate heat for friction welding at comparatively small pressures *σ*_3_.

**Figure 16:**
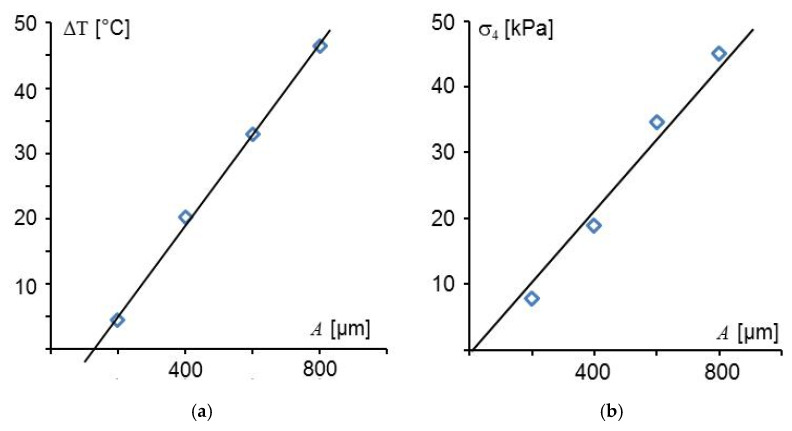
Temperature rise ΔT (**a**) and shear stress σ_4_ (**b**) as a function of the amplitude *A* of the linear vibrations of the friction welding machine.

Further measurements were performed at 2.6 MPa with different vibration amplitudes A. As shown in Figure 16b, the measured shear stress is a linear function of vibration amplitude. However, a straight line through the measured temperatures as a function of vibration amplitude seams not to go through the origin of the graph indicating that at small amplitudes there is no linear relationship between generated temperature and amplitude. Unfortunately, measurements could not be extended to smaller amplitudes because the friction welding machine only works with amplitudes larger than a minimum.

### 3.2. Circular Friction Welding Machine

**Figure 17:**
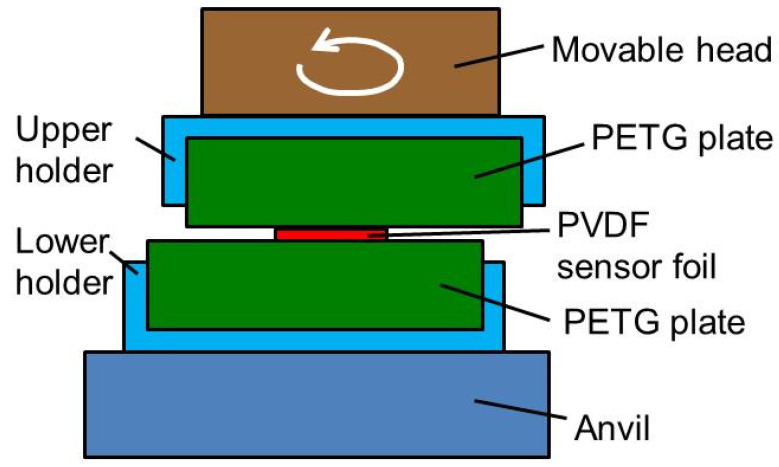
Schematic drawing of the experimental setup in a circular friction welding machine.

Shear stress and temperature change were also measured of a circular friction welding process performed with the machine ZMT 2.0 from Fischer Schweißtechnik (Berkatal, Germany). Figure 17 shows the setup of this experiment. A lower holder was mounted on an anvil. In this holder, there a square PETG plate, 200 mm and 4 mm in edge length and thickness, respectively, was fixed. A PVDF sensor foil was placed onto the PETG plate. On the upper part of the machine, there is a head able to perform circular movements with an adjustable radius in the range between 350 and 600 µm. Onto this head, there was mounted an upper holder in which another PETG plate with the same dimensions as the other one was fixed.

The machine exerted a pressing force of 100 N and started a circular movement with a radius and frequency increasing to up to 0.35 mm and 70 Hz, respectively. To avoid damage to the PVDF sensor, the parameters where chosen much smaller than usual for a real welding. The PETG plates were rubbed over the PVDF foil generating shear stress.

**Figure 18:**
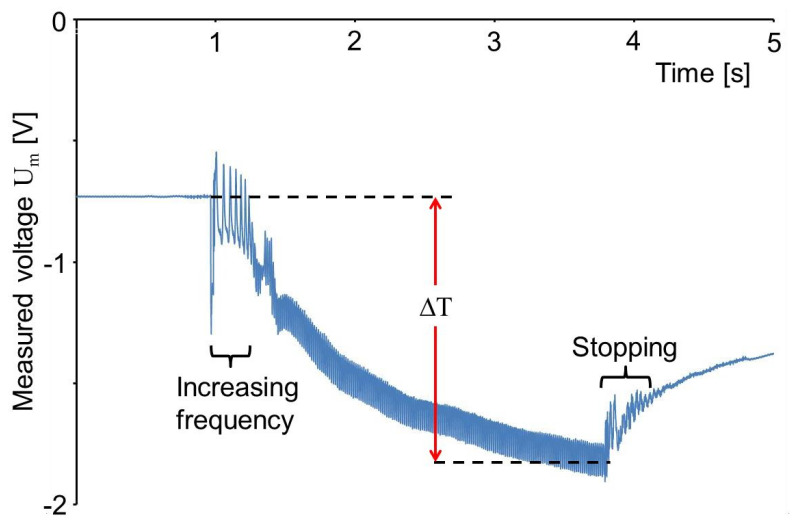
Voltage measured as a function of time by a PVDF sensor foil in a circular friction welding machine.

Figure 18 shows a measurement performed in the circular friction welding machine similar to the one with a linear friction welding machine shown in Figure 10. Contact stroke and pull back were not measured by the PVDF sensor foil because they occurred before and after the measurements were taken. The capacitor parallel to the PVDF sensor was C_0_ = 9.6 nF. Since the PVDF sensor foil generates a 10 times larger charge when sheared in 1-direction compared to shearing in 2-direction (cf. Figure 7a,b), an oscillation is seen in Figure 18 corresponding to shearing in 1-direction.

At the beginning, the vibration head moved in a circular movement to the radius of the movement, the acceleration of the movement caused large shear stress. After 500 ms, the circular movement became steady, so that the measured shear stress stayed constant. As a consequence, a steady voltage decrease due to friction heat was superimposed to the periodic voltage change. 2.9 s after it had started, the vibration head began to decelerate and move back to the circle center. The vibration stopped after 400 ms. The temperature rise (*ΔT* = 1 °C) was obtained from the voltage difference (−0.92 V, 10.5 nC) as shown in Figure 18.

**Figure 19:**
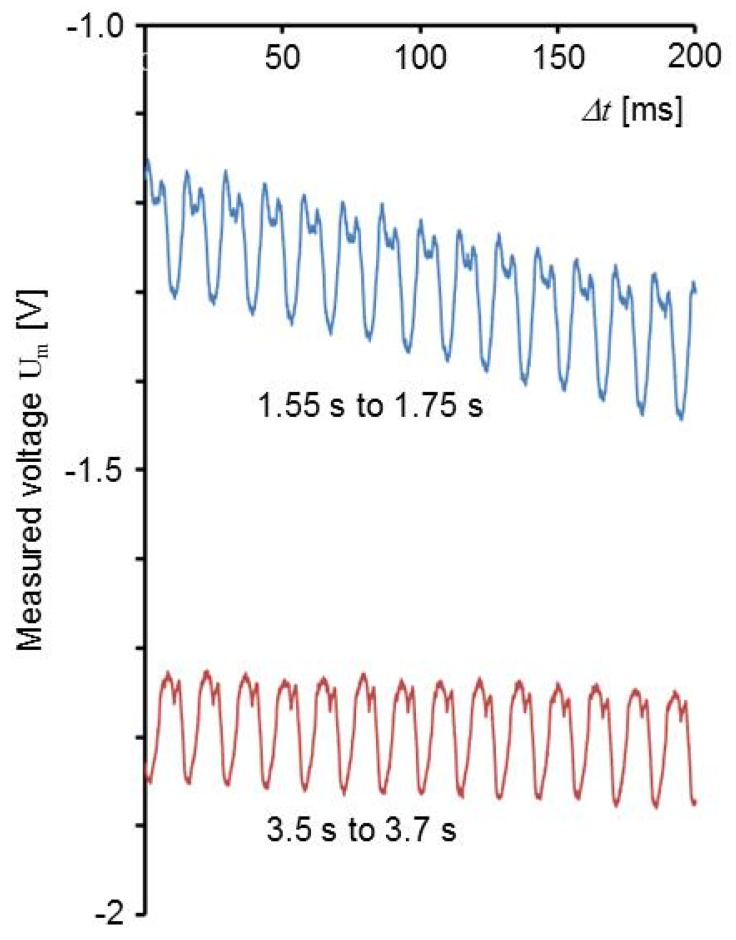
Voltage measured as shown in Figure 18 at begin and end of machine movement. The corresponding time range from Figure 18 is shown next to the curves.

Similar as in the case of the linear friction welding machine, the shape of the signal is changing with the duration of the lateral movements (Figure 19). It is not clear whether the observed non-sine-like shape is influenced by receiving an electrical signal from the electromagnetic drive in the head of the machine. Besides this, charge generated due to shear in 2-direction and deviations from a pure circular movement may have an influence as well as other vibrating parts of the machine. The shear amplitude *A*_4_ calculated from the data is 1.8 kPa corresponding to 0.07 V and 0.8 nC.

**Figure 20:**
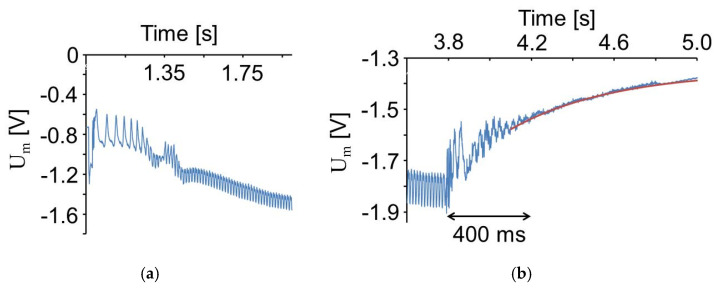
Voltage measured as shown in Figure 18 in the time range of start (**a**) and end (**b**) of machine movements. The curve adapted to the data according to Equation (11) is shown in (**b**).

Figure 20a shows the signal displayed in Figure 18 in the time range of the beginning of the circular movements of the machine head. The maximum shear component in 1-direction at the beginning is 11.4 kPa corresponding to 0.45 V and 5.1 nC. It is clearly seen that the speed of circular movement is increasing until a shear oscillation of 71 Hz corresponding to an angular velocity of 446 rad/s is reached after 500 ms.

The time range in Figure 18 of the stopping of circular movements is shown in Figure 20b together with the curve adapted to the data with Equation (11). It is recognized that the movements of the machine stopped approximately 400 ms after the drive was stopped.

The overall temperature change generated by friction indicated in Figure 18 is calculated to be 1 °C. This is by far not enough to melt the surface of the polymer. The parameters of the welding were chosen such to avoid damage to the PVDF sensor. The time constants θ of temperature rise and cooling after stopping the movements of the machine top have been found by adapting Equation (11) to the curve in Figure 18 to be 920 ms and 500 ms, respectively.

## 4. Discussion

**Figure 21:**
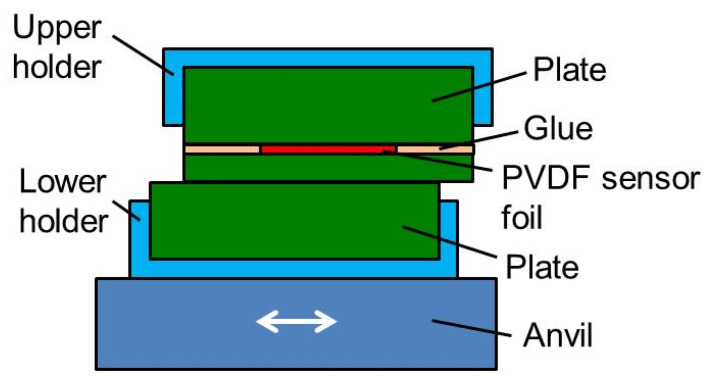
PVDF sensor foil glued between two plates.

To avoid damaging the PVDF sensor foils, the welding parameters used for the measurements shown in this paper were all chosen such that no real welding was achieved. The results show that PVDF sensor foils allow measuring shear and temperature at the interface of the bodies rubbed against each other. However, to draw reliable conclusions on the welding process, it is desirable to measure while a real welding is performed. Therefore for future investigations, it would be desirable to glue a sensor between two plates as shown in Figure 21. Due to the small heat conductivity of the plate below the sensor, the PVDF foil is thermally isolated from the area where the friction heat is generated. Less heat arrives at the PVDF foil and delayed. Thus the sensor foil is not overheated and shear stress and pressure rise are still recorded. On the other hand, it will be difficult to draw conclusions of the generated temperature.

For friction welding, it is important that the relative movement of the samples stops very quickly ensuring that a tight bond is established [14,15]. The polymer should not cool so much that it becomes hard before the movement is completely stopped because otherwise residual stresses are generated in the welding zone. Therefore, besides the stopping time, also the time constant of cooling is important. If stopping of the movements requires a longer time, cooling should be slower. This could be achieved by heating the upper and lower holder and work pieces. Another possibility is to install breaks in the friction welding machines. Measurements such as the ones described in this paper may help to find out how much the work pieces should be heated and how strong a break needs to be.

It is not possible to draw general conclusions from the results of the measurements presented in this paper because both stopping and cooling will be different if real samples are welded, and higher temperatures will be reached. Also the roughness of real samples will be different than the one of the PVDF foils used for the experiments presented in this paper. However, this paper explains how the behaviors of different friction welding machines can be compared and how it can be investigated to recognize the potential for improvements.

The authors of this paper are not able to explain the observed shape of the signal shown in Figure 13 and Figure 19 changing to a sine-like curve when the drive is stopping as shown in Figure 14. Several reasons may be taken into consideration such as an electrical signal generated by the electromagnetic drive which is caught by the sensor like an antenna or some vibrating part in the machine or the sound generated by the drive. In principle non-harmonic movements of the machine can be discovered by the PVDF sensor, but it needs to be made sure before, that the signal is really due to the relative movement of the work pieces. For the strength of the friction weld, this is not expected to be important, but it may be used as an indication of machine defects.

Another observation is that the charges generated on the PVDF sensor are a nearly linear function of the shear force in extrusion direction while shear in the direction perpendicular to this (2-direction) generates charges with the same sign no matter whether the force acts to the right or left. The anisotropic properties of a PVDF sensor are generated by stretching the foil during extrusion or afterwards and by applying a large electrical field at elevated temperatures. Thus, the dipoles of the polymer crystals are aligned in 1- and 3-direction. If after fabrication the sensor is sheared in 2-orientation, the same or a similar charge is generated independent on the direction.

In Figure 16a, it is seen that a straight line through the data points does not go through the origin. It cannot be proven by the measurements presented here, but it may be an indication that at small amplitudes heating is based only on shearing of the sample and not on friction heat. Friction heat is expected to increase proportional to the amplitude of relative movement, but heat generated by the elastic deformation of the polymer should be a quadratic function of the amplitude. A quadratic increase of temperature with small amplitudes would explain why the straight line does not go through the origin. Unfortunately, it was not possible to measure at smaller amplitudes.

## 5. Conclusions

If the effects of shear direction, compression and temperature on the measurements are carefully taken into account, time of pneumatic pressure rise, shear stress, generated temperature by friction heat, cooling times, contact stroke, acceleration and stopping times of movements, vibration or rotation frequency, and pull back time and force of a friction welding machine can be measured. This way the performance of different machines and indications for possible improvements are obtained.

Unfortunately, the sensitivity of PVDF sensor foils purchased from the same vendor at the same time may differ on the order of 20%. This limits the accuracy of absolute measurements of force, shear, temperature and pressure because every sensor foil needs to be calibrated. On the other hand, measurements of frequencies, times and relative measurements of forces, shears, pressures and temperatures are possible with higher precision. The accuracy of time measurements in the experiments described here was limited by the sampling rate of 100 kHz.

In Figure 15, it is shown that, at least at the beginning of friction welding, more heat and more shear stress are generated if the welding zone is compressed with less force. This indicates that with measurements like these the optimum pressure load for a quick welding process can be found.

The method presented here is not limited to friction welding but may enable measurements in a lot of applications where changes of temperature, shear, pressure and forces need to be known at the interface of bodies in close contact. One example is the new developed process friction embossing employed for the fabrication of micro structures from polymers [35].

## Figures and Tables

**Table 1:** Geometrical dimensions of PVDF sensor foils and electrodes.

	Length	Width	Thickness	Distance
Sensor foil	L_F_ = 41 mm	b_F_ = 16 mm	h_F_ = 58 µm	
Electrodes	L_E_ = 29 mm	b_E_ = 12 mm		h_E_ = 30 µm

**Table 2:** Piezoelectric moduli of force measurements and ratios of generated charge and stress.

Direction i	1	2	3
*g*_3i_ [V m/N]	0.19	0.062	−0.65
*d*_3i_ [pC/N]	22	7.4	−77
Q_3_/*σ*_i_ [nC/MPa]	8.0	2.6	−27

**Table 3:** Approximate slopes of the characteristic curves shown in Figure 7a and the moduli *d*_34_ calculated by solving Equation (7) as a function of temperature.

ΔT [°C]	20	26	32
*d*_34_ [nC/N]	1.3	2.1	2.7
Q_3_/*σ*_4_ [nC/kPa]	0.46	0.73	0.93
